# A device for surveillance of vascular access sites for bleeding: results from a clinical evaluation trial

**DOI:** 10.1038/s41598-020-74571-2

**Published:** 2020-10-23

**Authors:** Chang Yin Chionh, Desilyn Yuqing Soh, Chee How Tan, Jien-Yi Khaw, Ying Ching Wong, CREMERE Group, Debajyoti Malakar Roy, Sreekanth Koduri, Alvin Kok Heong Ng, Cheng Boon Poh, Chee Yong Ng, Wenxiang Yeon, Shaohui Foong

**Affiliations:** 1 Department of Renal Medicine, Changi General Hospital, Singapore, Singapore; 2 Clinical Trials and Research Unit, Changi General Hospital, Singapore, Singapore; 3 Engineering Product Development Pillar, Singapore University of Technology & Design, Singapore, Singapore

**Keywords:** Biomedical engineering, Electrical and electronic engineering, Kidney diseases, Acute kidney injury, Engineering, Adverse effects

## Abstract

Post-procedural wound haemorrhage is a potentially life-threatening complication. For haemodialysis patients, bleeding is often encountered after vascular access procedures and fatal episodes have been reported. Visual monitoring for bleeding is manpower intensive and bleeding episodes may still be missed between inspections. A device, Blood WArning Technology with Continuous Haemoglobin sensor (BWATCH), was developed to detect bleeding from wounds. This a prospective, observational clinical trial on patients who have had a dialysis catheter inserted or removed. The battery-powered, disc-shaped device (43 mm diameter, 12 mm height) was placed over the dressing for at least six hours. The device detects reflected light with characteristics specific for haemoglobin and an alarm would be triggered if bleeding occurs. There were 250 participants (177 post-insertion, 73 post-removal) and 36 episodes of bleeding occurred. The device alarm was triggered in all instances but there were also 9 false alarms. Specificity was 95.8%, false positive rate was 4.2% and positive predictive value was 80.0%. Sensitivity and negative predictive value were 100% but detection failure may still occur due to improper application or device maintenance. The use of technological aids for monitoring improves patient safety and may reduce demand on manpower.

## Introduction

Post-procedural wound haemorrhage is a potential complication following any invasive procedure. Despite care taken to mitigate the risks, bleeding complications inevitably occur with varying degrees of severity. While data on such complications are rarely available in public domain, a published audit noted a post-operative bleeding rate of 6.3%^1^. In the setting of haemodialysis, with multiple repeated vascular access procedures performed on uremic patients prone to bleeding^2^, post-procedural bleeding may be even more common.

A few years ago, through personal communications, a few incidents of catastrophic bleeding following removal of vascular dialysis catheters were flagged up for review. Published literature on this complication was scant. Serious adverse events following central venous catheter removal, while rare, were unlikely to be isolated incident^3^. The most comprehensive report in public domain came from the British National Reporting and Learning System, with 6 incidents of late bleeding following femoral line removal documented over 3 years. Of these incidents, 3 resulted in deaths and 2 suffered more than one litre of blood loss^4^.

The UK Renal Association, British Renal Society and Intensive Care Society subsequently released a recommendation for the safe removal of a temporary femoral dialysis line in 2019^5^. It was recommended that the frequency of observations for bleeding should be determined based on level of risk. Protocols implemented in some units required an intense period of monitoring every 5–15 min in the first 2 h^6,7^.

However, such frequent monitoring strategies are highly manpower intensive and places further demands on already scarce nursing manpower. The shortage of nurses globally has been termed as a global crisis since 2002 by the International Council of Nurses^8^ and the problem remains acute today^9^. Delegating monitoring tasks to attendants may not be safe or permitted in many jurisdictions, especially when the potential risk of a missed bleeding episode is death.

As catastrophic bleeding is rare, this may also lower the level of watchfulness and awareness. It has been shown that infrequently occurring monitoring targets are more likely to be missed than more frequently occurring counterparts^10,11^. It is difficult to expect healthcare staff to maintain a consistent high level of vigilance for a low probability event. Coupled with multiple demands for attention, the possibility of human error and missed catastrophic bleeding becomes higher, particularly if the patient is incapacitated and unable to call for assistance.

We searched for a technological solution to safely monitor patients for bleeding following catheter removal. Commercial systems^12,13^ and products in development^14^ were available. The products detected the presence of fluids by changes in physical properties such as electrical resistance^13^, capacitance^14^ or opacity to light^12^. However, these detection methods were not specific for blood. The users’ preference was for a device which could specifically detect bleeding without direct contact with the wound or blood. Other requirements included ease of use, wireless and compact form factor. Ideally, it should also be suitable for monitoring other wounds at risk of bleeding, for example, following arterial access for coronary angiogram or vascular interventions.

**Figure 1:**
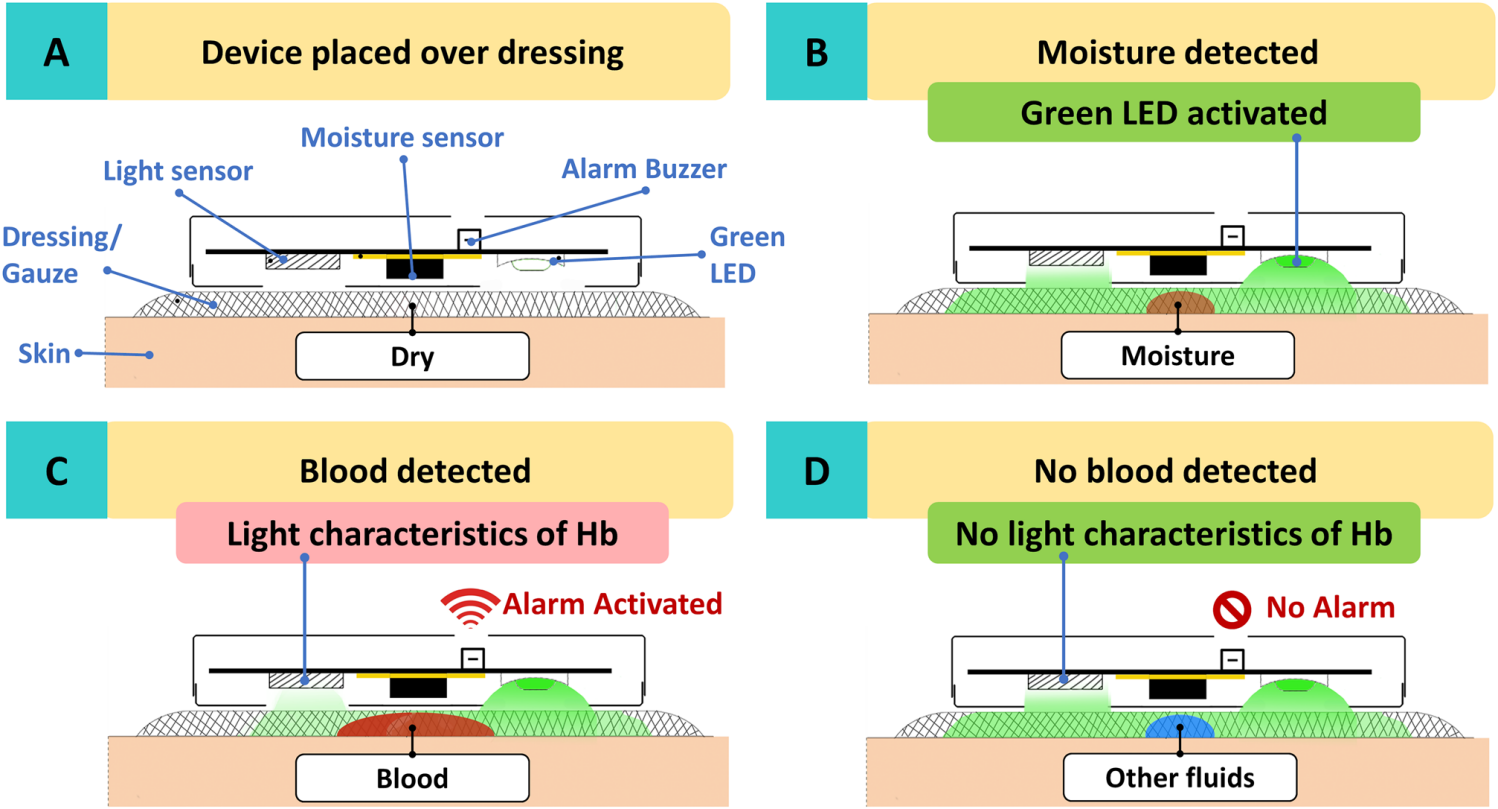
Illustration of the technical process of monitoring and detection of blood by the BWATCH device. (**A**) Device placed over a dressing. (**B**) When moisture is present in the dressing, a change in capacitance is detected and the green light emitting diode (LED) is activated. (**C**) Haemoglobin (Hb) in the blood absorbs 525 nm wavelength light. When the light sensor does not detect 525 nm light, the alarm is triggered. (**D**) With other fluids (non-blood), the light sensor detects 525 nm light and no alarm is triggered.

Through a research collaboration between the hospital and a technological university, supported by a national innovation grant, a device was developed to try to meet the specified requirements. The technological concept was presented in engineering and technology conferences^15,16^. In brief, the device is a stand-alone, disc-shaped device (43 mm in diameter, 12 mm thick, weight 11.5 g). The device is powered by an in-built rechargeable battery which provides a runtime range between 7 to 9 h with each charge. The device is placed over bandages or wound dressings and continuous monitoring takes place. The underlying detection principle is based on the light absorption properties of haemoglobin, which is most significant at the 525 nm wavelength^15^. When the sensor detects the absence of 525 nm wavelength light, it triggers a loud alarm, alerting medical staff to the patient. Figure 1 summarises technical process of monitoring and blood detection.

**Figure 2:**
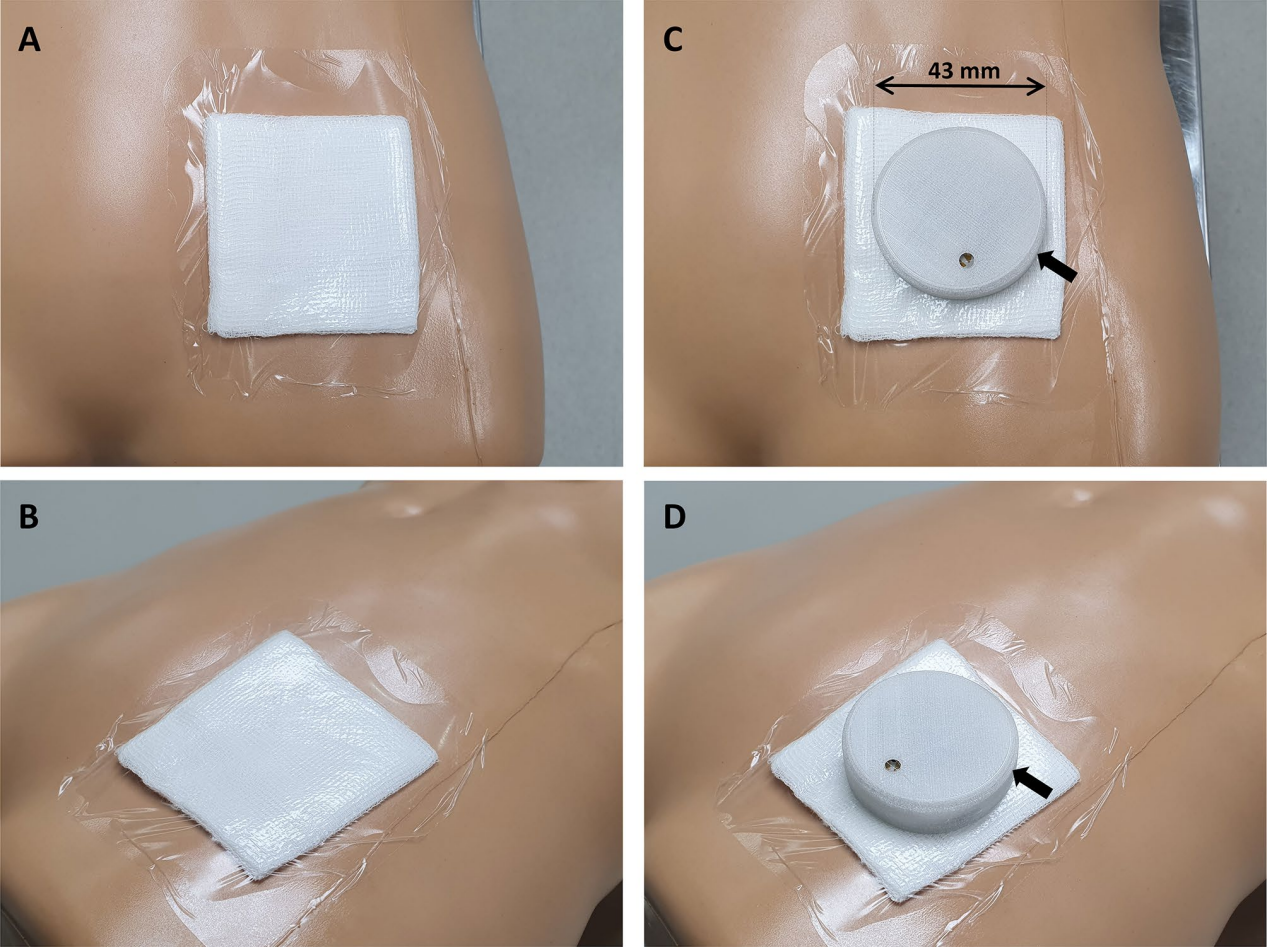
Images showing how the device is applied, simulated on a mannequin. Image (**A**) and (**B**) shows the dressing applied. Image (**C**) and (**D**) shows the device placed in-situ, indicated by black arrows.

The device can be used over transparent plastic dressings and does not require direct contact with blood (see Fig. 2). Laboratory tests of the prototype performed with human blood at haemoglobin levels ranging from 3 to 12 g/dL demonstrated a 100% detection over 120 experimental runs. Components of the device are commercially available and a limited production of 10 devices cost USD 60 per unit.

The prototype, Blood WArning Technology with Continuous Haemoglobin sensor (BWATCH), was evaluated in this clinical trial. The primary aim of this study was to report on the sensitivity of the device in detecting bleeding in a clinical environment. The secondary aim is to determine the specificity and false positive rate of the device for actual bleeding.

## Results

Of the 413 eligible patients, 252 consented for the study between 01 March 2019 to 30 June 2020. Two patients, planned for tunnelled catheter insertion, subsequently withdrew from participation before monitoring started and were not included; as an ethical requirement, all participants can withdraw participation at any time without obligation to provide a reason. The remaining 250 patients completed the monitoring protocol. Recruitment was suspended between 7 April to 1 June 2020 as all research activities were suspended and resources were diverted as part of the institutional and national response for the COVID19 pandemic.

**Figure 3:**
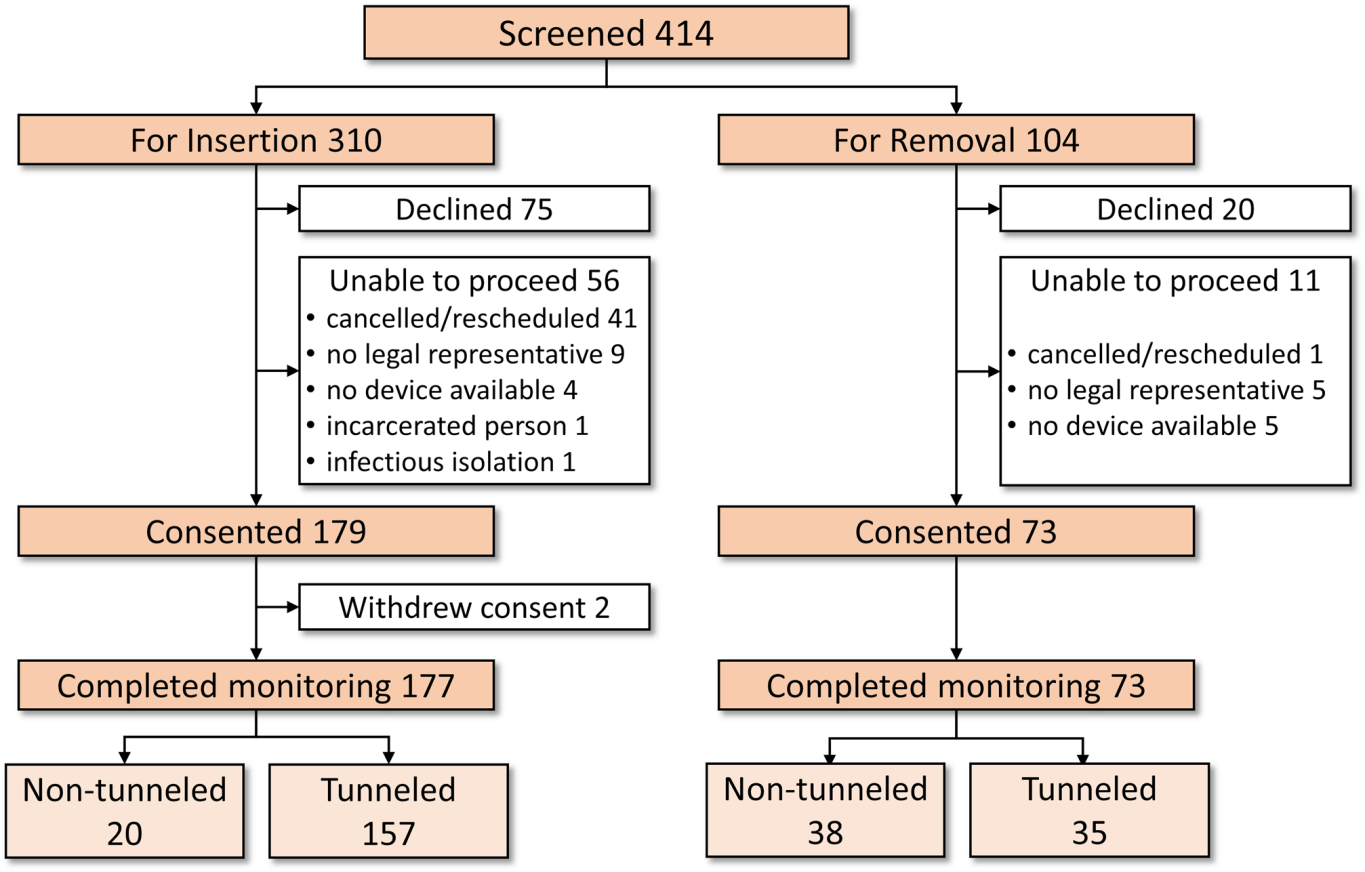
Flow diagram of the recruitment of participants for study and completion of study protocol.

A hundred and seventy-seven patients were monitored following catheter insertion, of whom 157 had a tunnelled dialysis catheter inserted while the remaining were non-tunnelled. Seventy-three were monitored following catheter removal, of whom 35 had a tunnelled dialysis catheter. A flow diagram of patient recruitment is shown in Fig. 3.

There were 110 (44.0%) females and the median age of the participants was 65 (55–73) years. As this study recruited patients on dialysis, uremia was a significant risk factor for bleeding, with a median urea of 21.6 (15.5–27.3) mmol/L. Otherwise, the coagulation profile of this cohort was of low risk for bleeding with a median international normalized ratio (INR) at 0.99 (0.95–1.07), partial thromboplastin time 29.9 (26.9–33.6) seconds and average platelet count 209 (159–274) × 10^3^/μL. Detailed baseline characteristics of the participants are listed in Table 1.

**Table 1:** Characteristics of participants.

Characteristic	All (n = 250)	Reference range
Insertion of non-tunnelled catheter	20 (8.0%)	–
Insertion of tunnelled catheter	157 (62.8%)	–
Removal of non-tunnelled catheter	38 (15.2%)	–
Removal of tunnelled catheter	35 (14.0%)	–
Days before removal (non-tunnelled)	5 (4–7)	–
Days before removal (tunnelled)	98 (22–208)	–
Sex (female)	110 (44.0%)	–
Age (years)	65 (55–73)	–
Serum urea (mmol/L)	21.6 (15.5–27.3)	2.8–7.7
Serum creatinine (umol/L)	608 (439–817)	Females: 50–90; males: 65–125
eGFR (mL/min/1.73m^2^)	7 (5–9)	> 90
International normalized ratio	0.99 (0.95–1.07)	0.8–1.1
Partial thromboplastin time (s)	29.9 (26.9–33.6)	30–40
Platelet count (× 10^3^/μL)	209 (159–274)	140–400

Quantitative variables are presented as median (interquartile range); qualitative variables are presented as n (%).

*eGFR* estimated glomerular filtration rate.

There were 36 episodes (14.4%) of bleeding. Predominantly, this occurred after tunnelled dialysis catheter insertion (31 patients, 86.1% of bleeding instances). Bleeding was less common following removal of catheters, with 2 (5.6%) occurring after removal of non-tunnelled catheters and 3 (8.3%) after removal of tunnelled catheters.

There were no false negatives; all instances of bleeding were detected by the device. In addition, the device alarm was the first indication that bleeding has occurred in all cases; manual inspection did not detect any bleeding before the device alarm was triggered. In most cases, blood loss was minimal with partially blood-stained bandages. There were no catastrophic bleeding episodes with haemodynamic instability, requiring fluid resuscitation or blood transfusion.

In one instance, a small amount of blood was seen tracking along the external segment of a newly inserted femoral dialysis catheter. The catheter had been manipulated due to poor catheter flow during dialysis, and the bandage had been almost fully peeled off before being reapplied. Subsequently, the bandage dislodged due to loss of its adhesiveness and bleeding which occurred following manipulation did not stain the bandage. This was not considered a false negative as no blood was in the area of device surveillance but this highlighted the importance of proper application of bandage and device.

False positive alarms occurred in 9 instances. Of note, in all but one instance, the site being monitoring was in the chest or neck following internal jugular catheter insertion or removal. The device was likely activated when moisture (e.g. perspiration) was detected in the dressing. Although, no blood was present, there was possible leakage of ambient light which resulted in alarm activation. Ambient light leakage was less likely in the femoral site as it was usually covered under clothing and blankets.

Overall, for the detection of bleeding, the sensitivity and negative predictive value was 100% in this cohort with 250 surveillance episodes. The false positive rate was 4.2%, with a specificity of 95.8% and positive predictive value of 80.0%. Table 2 presents the full contingency table and error matrix for device response vs bleeding occurrence.

**Table 2:**
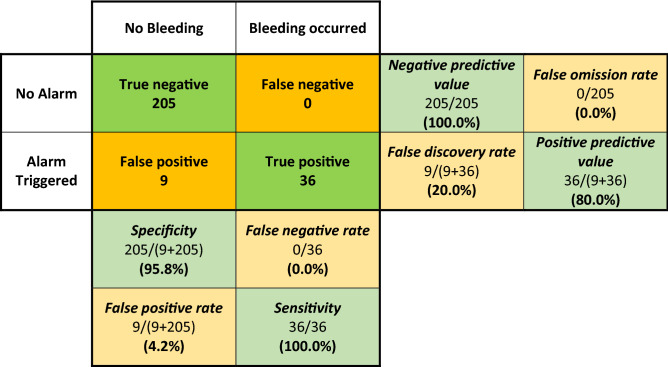
Contingency table and error matrix for device alarm vs bleeding occurrence.

## Discussion

In this report, we present data on the sensitivity and specificity of a novel bleeding detection device. The use of the device for continuous monitoring of wound sites for bleeding is expected to be safer with a lower potential for missed bleeding as compared to visual inspection at intervals. In our laboratory simulation of a slow oozing wound (30 mL/min), the device picks up the presence of blood within 4.88 ± 0.85 s from the onset of bleeding^17^.

The deployment of this device may reduce reliance on manpower and releases healthcare staff for other essential duties. During this clinical trial, routine intensive monitoring by nurses occurred concurrently as a safety assurance. The bleeding episodes in this study were minor and would not have required intervention. It could be said that the device may have inadvertently increased the time spent attending to minor bleeding episodes, but this must be balanced against the need to detect a catastrophic haemorrhage early. As we gain confidence from wider use of this device, the frequency of visual inspections and manpower requirements may be reduced with no compromise to safety.

An important technological aspect of this device is that it does not require direct contact with the wound or blood. Detection can occur across clear plastic and the device itself was sheathed in a disposable polyethylene cover. This is important for infection control and preventing cross contamination.

As part of product development, the research team undertook a risk analysis and the potential risks identified were largely related to technical failure due to improper application or maintenance. For example, the device may dislodge during the monitoring period if it is not applied securely, or if the user fails to check the indicator lights to ensure the device is switched on or adequately charged. The incorrect application of the dressing is a significant risk, with one case as reported above illustrating this issue. The bandage, which was reapplied after removal, had lost its adhesiveness and the system could not work without the bandage coming in contact with blood.

The application of the device is simple but the user must still be familiarized with its use and proper application prior to deployment in the ward. Only if used correctly can the device reduce the risk of undetected bleeding and its sequelae.

Other disadvantages include a false discovery rate of 20% of all alarms triggered. While the number of false alarms may be reduced by increasing the threshold of detection, we decided against this as it may reduce the sensitivity. While false alarms bring inconvenience to the patients and healthcare staff, a missed bleeding may be potentially catastrophic and should be avoided at all cost. The overall impact of false alarms was low as out of 250 monitoring sessions, staff had to attend to false alarms in 9 instances, which represents a false positive rate of 4.2%.

One hypothesis on the cause of the false alarms was that flexing and stress on the electrical components of the prototype device could result in electrical noise and activate the sensor which was mounted on a flexible printed circuit board. In the last 45 cases, a more rigid casing was utilised with no further false alarms while 7 true bleeding episodes detected.

The leakage of ambient light was identified as another potential cause of false alarms. Artificial lighting sources such as LEDs or halogen/incandescent bulbs may have higher peaks in the violet-blue spectrum (400–490 nm) or yellow–red spectrum (570–780 nm), while the device is activated when 525 nm light levels are low. Potentially, false positives could be avoided by programming the device to remain silent if a wide spectrum of light is detected. On the other hand, the triggering of alarm by ambient light could be a potential safety feature in the event the device is partially dislodged and exposed to environmental light. As such, we did not alter the detection protocol in this aspect.

A more precise method to address this issue of false alarms will require collection of more data. Variations in light leakage could be recorded across a range of pressure applied on the device and bandage, and in different lighting conditions. With more data, advanced processing of the light sensor readings could allow better differentiation of true haemoglobin presence vs ambient light leakage, accounting for different degrees of light leakage which depends on how tightly the bandage is applied.

Currently, only auditory alarms are built into the device. This may limit its usage in noisy environs or in isolation rooms. However, it is possible to add on components with wireless communication capabilities (e.g. Bluetooth Low Energy, IoT networks) to transmit a signal to a central system, such as a nurse call system.

It is important to note that this device has only been tested on dressings with a white background. Dressings of other colour may alter the intensity of light of various wavelengths reflected from blood. It is developed only for the detection of external bleeding and does not detect internal haemorrhage. However, in our review of the literature, severe morbidity or death was reported only with external haemorrhage following catheter removal.

Further development will include extension of use in a wider group of patients in order to gain more clinical experience and confidence in the sensitivity of the detector. In the dialysis setting, the device can be used to monitor for blood loss from dialysis line or venous needle disconnection which had also been identified as a significant risk to patients^4,18,19^. Although no bandage or gauze is usually placed over the dialysis needle site, the pooling of blood underneath an occlusive plastic dressing can still be detected by the same principles. However, the current shape of the device may not be ideal for placement over a cannulated fistula or graft. The form factor should be altered to fit this purpose and further clinical trials are required.

Indications for use can also be expanded to other wounds or locations which may be prone to external bleeding, for example post-cardiac catheterization or other percutaneous radiological interventions. The device may also be tested in other clinical situations outside of the hospital, such as field monitoring of traumatic wounds. The device cost is low and expected to be even lower with mass production. The estimated cost is less than USD 15 for a production order of 1000 units and it can be used for up to 500 cycles (based on the rated number of charge cycles of the lithium-ion battery).

In conclusion, the reliability of the BWATCH blood detection device is demonstrated in this clinical study. Undetected heavy bleeding, while rare, has been associated with catastrophic outcomes. It is challenging to maintain long-term vigilance for infrequently occurring catastrophic events and the use of technology for monitoring improves patient safety.

## Methods

This trial was first registered prospectively with the institutional review board and ethical approval was obtained on 07 November 2014 (SingHealth Centralised Institutional Review Board, Reference No.: 2014/2036). The study plan was also submitted to the national funding agency on 01 February 2018 (NHIC-I2D-1608124). A revised study protocol was submitted to the institutional review board and ethical approval was obtained on 28 May 2018 and patient recruitment started on 01 March 2019. The study protocol is available at ClinicalTrials.gov (NCT04285775). All research was performed in accordance with relevant guidelines, regulations and national human biomedical research legislation. Informed consent was obtained from all study participants or their legal representatives if cognitively impaired.

This a prospective, open-label observational study performed in an acute care hospital. We recruited inpatients planned for dialysis catheter insertion or removal, based on standard clinical care and indications as identified by the primary nephrologist-in-charge. Patients who declined participation or patients who were unable to provide consent and had no legal representatives were excluded.

Dialysis catheter insertion was added as an inclusion criterion because delayed bleeding following insertion may be more common and this would increase the number of bleeding events for detection by the device. Assuming the rate of bleeding to be 15% based on past observations, for a 95% level of confidence and a precision of 5%, a sample size of at least 196 participants would be required.

All procedures were carried out by credentialled doctors from the Department of Renal Medicine or Interventional Radiology. All catheter insertions were carried out under direct ultrasound or fluoroscopic guidance under local anaesthesia. Local anaesthesia was also applied for removal of tunnelled catheters but no anaesthesia is required for removal of non-tunnelled catheters. The gauge of the catheters used was 11.5 Fr for non-tunnelled catheters and 14.5 Fr for tunnelled catheters.

The procedurist would secure haemostasis following the procedure and apply a bandage. Two types of bandages were used according to procedurists’ preference—a cotton gauze overlaid with a transparent polyurethane dressing (Tagaderm, 3 M Health Care, Minnesota, USA) or an absorbent, self-adhesive dressing (Mepore, Molnlycke Health Care AB, Gothenburg, Sweden). The investigators would then place the device over the dressing and secure it in place with another layer of transparent polyurethane dressing. The device was then left in-situ for at least 6 h. The device must be charged before each use—a green LED indicates that the device is charged while a red light indicates inadequate battery levels.

Throughout the observation period, routine monitoring and inspection for bleeding by the nurse-in-charge would continue as per hospital clinical protocol—15-min intervals in the first hour, 30-min intervals in the second hour and hourly from the third to sixth hour. Upon removal of the device, the bandage was inspected for visible blood stains.

Alarms triggered or bleeding episodes were documented in a case report form. A false positive was defined as an alarm triggered with no fresh blood stains on the bandage on visual inspection. A false negative was defined as a failure to trigger an alarm when blood stains were found to be present in the bandage, regardless of size or intensity of stains.

Baseline demographic data and relevant laboratory indices of coagulation profile and renal function were collected. Continuous data were represented by median (interquartile range). Frequency of discrete events were expressed as number of events and percentage. From the bleeding incidences and device alarm activation rates, the sensitivity and specificity of the device for detection of true bleeding were calculated. Data was processed using IBM SPSS Statistics version 20 (IBM Corporation, Armonk, New York, USA).

The manuscript was prepared according to STROBE reporting guidelines^20^.

## Data Availability

The datasets generated during and/or analysed during the current study are available from the corresponding author on reasonable request.
